# Development and Validation of a Chinese Version of a Professional Identity Scale for Healthcare Students and Professionals

**DOI:** 10.3390/healthcare8040451

**Published:** 2020-11-01

**Authors:** Hung-Chang Liao, Ya-huei Wang

**Affiliations:** 1 Department of Health Services Administration, Chung Shan Medical University, Taichung 40201, Taiwan; hcliao@csmu.edu.tw; 2 Department of Medical Education, Chung Shan Medical University Hospital, Taichung 40201, Taiwan; 3 Department of Applied Foreign Languages, Chung Shan Medical University, Taichung 40201, Taiwan

**Keywords:** professional identity, healthcare, exploratory factor analysis, confirmatory factor analysis

## Abstract

This study was intended to develop a comprehensive and psychometrically adequate professional identity scale for healthcare students and professionals based on Taiwanese cultural contexts. In order to elicit a more consistent result of the psychometric indices of the newly developed scale, the study adopted a combination of exploratory factor analysis (EFA) and confirmatory factor analysis (CFA) to examine the consistency of the scale factors. In a pilot study of 562 randomly selected healthcare students and professionals, the EFA yielded a 33-item four-factor model with the terms “professional commitment & devotion” (16 items; 47.33% of variance), “emotional identification & belongingness” (7 items; 9.72% of variance), “professional goals & values” (5 items; 8.17% of variance), and “self-fulfillment & retention tendency” (5 items; 3.38% of variance). The CFA yielded an 18-item four-factor model with good or even excellent fit to the data, where the χ^2^/df ratio = 1.138, Tucker Lewis index (TLI) = 0.997, comparative fit index (CFI) = 0.997, and root mean square of approximation (RMSEA) = 0.016. The convergent validity and discriminant validities were also conducted to test the feasibility of the Professional Identity Scale for Healthcare Students and Professionals (PIS-HSP) scale. For the EFA model, the Cronbach’s alphas for the four factors and the overall scale ranged from 0.84 to 0.96; for the CFA model, the Cronbach’s alphas and composite reliabilities were, respectively, in the ranges of 0.78–0.93 and 0.78–0.97, demonstrating satisfactory reliabilities. The results proved that the developed PIS-HSP can be a reliable measurement tool to assess professional identity for healthcare students and professionals.

## 1. Introduction

While “identity” refers to people’s perception of who they are, what kind of people they like to be, and how they are connected to others [1], “social identity” refers to people’s self-perception of what social group they belong to in connection with certain values and emotional attachments to that group [2]. “Professional identity,” as one form of social identity, refers to people’s professional perception of themselves based on their values and beliefs, which guide the way they think, behave, and interact with social and professional norms [3,4]. Adams et al. [5] defined professional identity as the attitudes, values, knowledge, beliefs, and skills shared with others within the same professional group. It is also one’s perception as a professional sharing a set of beliefs, attitudes, and knowledge, and one’s understanding about one’s role within the work context [5,6]. Shared identity can also help professionals to differentiate themselves from other professional groups in the work context [5,7]. In addition, those having stronger identification with their professional group have greater job performance, satisfaction, and retention [8].

Professional identity among healthcare professionals refers to one’s awareness of being a professional in the healthcare field and one’s identification with healthcare groups and contexts that one belongs to by virtue of one’s occupation or career. The formation of professional identity involves an intra-individual process in which individuals are not only motivated but are cognitively prepared to employ their expected professional talents and competencies [9]. Moreover, it involves a formation of “collective identity” [10], in which individuals have to share certain attitudes, values, knowledge, beliefs, and skills within the same professional group, hence differentiating themselves from other professional groups in the work context [5].

Kroger and Marcia [11] noted that the construction of professional identities is crucial in the transition from adolescence to adulthood. Therefore, college students have to devote much more effort to exploring what they want their future profession to mean to them and who they want to be—while simultaneously pursuing the professional knowledge necessary to achieve those goals [12]. Via the exploration of professional identification, they may determine that they are ready to make a commitment to their chosen profession [13]. Research has shown that the professional identity of healthcare students is socially constructed [6,14]. The way medical care students understand their professional boundaries and the way they interact with patients or colleagues influence their expectations, actions, and judgment in their profession, as well as their future professional lives [15]. In healthcare, professional identity can somehow soothe the negative effects of a high-stress workplace. Those healthcare professionals with a stronger professional identity can not only benefit themselves, but also positively impact their patients and coworkers [16,17], because they know how to manage their professional roles. They can perceive themselves as a professional under a set of shared attitudes, values, and beliefs within the same professional group [5,6]. In addition, while thinking, acting, and interacting with patients within social and professional norms [3,4], they will have less difficulty in communicating with co-workers and patients. Hence, somehow, their work stress can be relieved.

The way healthcare professionals perceive themselves influences what kinds of choices and judgments they make and, therefore, impacts their professional attitudes, values, and commitment in healthcare contexts. Healthcare professionals’ and students’ professional identities are socially constructed by their social interactions with others in the healthcare workplace [16]. Therefore, they know how to interact with patients and patients’ families. Moreover, they may realize the boundaries of the healthcare profession and, hence, learn how to interact with other healthcare professionals to facilitate interprofessional healthcare teamwork [6]. Thus, healthcare professional identity may be defined as a set of beliefs, attitudes, and understanding about one’s role in the work context and healthcare system [6,14].

However, in the healthcare service, there are still differences in the responsibilities tied to different disciplines. Certain professionals in the same discipline, such as in the disciplines of medicine, nursing, social work, medical laboratory technology, or hospital administration, may share the same disciplinary responsibilities, which are quite different from those of other healthcare disciplines, thereby leading to different professional identifications in the healthcare service, albeit under the roof of healthcare. Each single professional discipline shares certain conceptualizations of professional identity. For example, those sharing the profession of doctor share the same conceptualization of professional identity. However, doctors, nurses, social workers, and counselors, though all working in the healthcare service, share different conceptualizations of professional or disciplinary identities and, hence, may have difficulties while addressing crucial healthcare issues in the complicated healthcare system.

Thorne [18] mentioned that, generally, medical professionals are initially resistant to change; however, while perceiving their professional boundaries, they may negotiate and adapt to the existing medical and healthcare contexts. Moreover, while realizing their professional boundaries, they may cross intra-professional boundaries and move toward interprofessional boundaries to redefine and reinterpret their interprofessional identity. Thus, through exploring their intra-professional and interprofessional identification in the healthcare system, they can later collaborate with their colleagues, both intra-professionally and inter-professionally, in different disciplines. In other words, healthcare professionals need to have not only an intra-professional identity, but also an interprofessional identity in order to facilitate collaboration and deliver integrated care to patients [19]. With dual professional identities, healthcare professionals are able to smoothly integrate various facets of their professional lives and manage potentially difficult clinical or healthcare situations involving interprofessional collaboration with greater ease.

Although professional identity development is an important concept in healthcare education, the process has not been well investigated from the perspective of collective healthcare professionals. Professional identity scales measuring a specific healthcare or medical care group may be informative in assessing the professional identity in that certain healthcare group; nonetheless, these scales, tailored to a certain group, are not appropriate to measure the collective professional identity among an interprofessional community of healthcare professionals. Therefore, in order to facilitate healthcare workers’ interdisciplinary collaboration and to identify themselves as a cohesive community within the healthcare system, healthcare professionals or students must have a shared professional identity, or a dual identity, through which they can maintain interprofessional roles while keeping their specific professional roles. In other words, through shared identity, healthcare professionals can identify with their specific professional groups, interdisciplinary communities, and the healthcare system as a whole [20,21].

## 2. Methodology

### 2.1. Procedure and Participants

This study was intended to develop a comprehensive and psychometrically adequate professional identity scale among healthcare professionals or students based on Taiwanese healthcare and medical contexts. In order to develop a Professional Identity Scale for Healthcare Students and Professionals (PIS-HSP), the researchers used the following steps: 1. item generation through systematic literature review and panel discussion; 2. initial scale item selection via theoretical analysis and expert review to confirm face and content validity; 3. data collection, using pilot questionnaires to examine psychometric properties; 4. item derivation and factor extraction; 5. internal consistency testing; and 6. validity and reliability testing.

In the first step of item generation, the researchers systematically reviewed the papers on professional identity from the four databases: EBSCO, Proquest, Pubmed, and ScienceDirect, as the theoretical basis for the generation of the initial item pool [22]. After item generation, a panel of experts in different disciplines met for the theoretical analysis of content validity to ensure whether the item pool reflected the desired theoretical construct [23] and to examine whether the item statements were appropriate or too difficult to understand [24]. The panel included three faculty experts in healthcare and medical education, instrument development and assessment, social sciences, and statistics. After three runs of expert panel discussions, the initial 95 items were reduced to 87 items, on a nine-point Likert scale (i.e., 9: agree extremely; 8: agree very much; 7: agree moderately, 6: agree slightly; 5: neither agree nor disagree; 4: disagree slightly; 3 disagree moderately; 2: disagree very much; 1: disagree extremely). The higher the score, the stronger the professional identity a participant had. The scale was initially developed in Chinese and then translated into English and later reviewed by two bilingual English teachers. It was then translated back into Mandarin Chinese. The original and subsequent versions were compared by two Chinese researchers with a master’s degree or Ph.D. degree in English; minor modifications were made to reach consensus. For content validity, the final version was once again checked by three experts and three university students to clarify each item.

Once the scale was developed, a pilot study was administered to 562 randomly selected healthcare students and professionals through on-site recruitment to test the validities and reliabilities of the assessment, including construct validity, internal consistency of the scale, expert validity, Cronbach’s alpha reliability, and so forth. Surveys returned with missing data were regarded as invalid surveys, resulting in a 90.57% response (*N* = 509) from the available participants. Of the 509 valid surveys, there were 339 women (66.60%) and 170 men (33.40%). The age range was from 19 to 58 years of age. This study was approved by the Institutional Review Board of Chung Shang Medical University Hospital (No. CS16157), following the guidelines of the Declaration of Helsinki [25]. In addition, the collected data from the participants were blinded, and all data were identified using numbers.

### 2.2. Statistical Analysis

In order to elicit a more consistent result on the psychometric indices of the new developed scale, the study adopted a combination of exploratory factor analysis (EFA) and confirmatory factor analysis (CFA) to examine the consistency of the scale factors [26]. The study first used SPSS (version 14.0) to perform EFA to explore the possible underlying factor structure of the developed PIS-HSP using the Kaiser-Meyer-Olkin test (KMO-test) and Bartlett’s test of sphericity to examine the sample size adequacy for EFA. The study also used Cronbach’s alpha to test the internal reliability within each factor of the scale; Pearson’s correlation coefficient between any two factors was also calculated. The researchers later used AMOS (version 24.00, IBM, NY, USA) to perform CFA to verify the factor structure of the extracted factors.

## 3. Results

### 3.1. Participants’ Demographic Characteristics

Table 1 presents some demographic characteristics of the valid surveys.

### 3.2. Exploratory Factor Analysis (EFA)

#### 3.2.1. KMO Test and Bartlett’s Test of Sphericity

**Figure 1:**
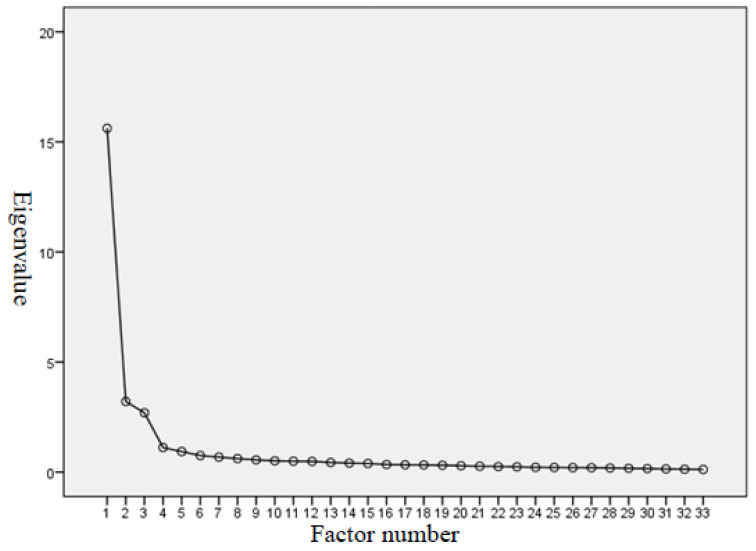
Scree plot for exploratory factor analysis on PIS-HSP.

The study used the KMO test and Bartlett’s test of sphericity to test the PIS-HSP scale’s structure validity, yielding the KMO value as 0.964, higher than the threshold value of 0.6 [27,28]. Bartlett’s test of sphericity is significant (approx. chi-square = 14,417.878; degree of freedom = 528; *p* = 0.000 < 0.05). KMO’s and Bartlett’s test results indicated the adequacy of the sample size for meaningful exploratory factor analysis. The scree-plot graphic also showed that four is the optimum number of factors for the PIS-HSP scale (see Figure 1).

#### 3.2.2. EFA Model for the PIS-HSP Scale

EFA was conducted to test the construct validity and the internal consistency of the scale, using eigenvalues of 1.0 and principal component analysis and a varimax rotation, which is an orthogonal rotation method commonly used for factor analysis [29]. An item was retained if it loaded greater than 0.60 on the relevant factor and less than 0.60 on the nonrelevant factor.

After conducting the EFA and principle component analysis, four factors and 33 items were identified for the EFA model for the PIS-HSP scale: “professional commitment & devotion,” “emotional identification & belongingness,” “professional goals & values,” and “self-fulfillment & retention tendency.” The four factors retained in the PIS-HSP accounted for 68.60% of the variance (see Table 2). Factor 1 contained 16 items related to “professional commitment & devotion” and accounted for 47.33% of the variance explained. Factor 2 contained 7 items related to “emotional identification & belongingness,” accounting for 9.72% of the variance. Factor 3 contained 5 items on “professional goals & values,” accounting for 8.17% of the variance. Factor 4 contained 5 items on “self-fulfillment & retention tendency” and accounted for 3.38% of the variance. All of the factor loadings were higher than 0.6, in the range between 0.61 and 0.89. The eigenvalues of the four factors from principle component analysis were all larger than one: 15.62, 3.21, 2.70, and 1.12, respectively (see Table 2). These results supported the uni-dimensionality of the PIS-HSP.

#### 3.2.3. Reliability of the EFA Model for the PIS-HSP Scale

Cronbach’s alpha was used to test the stability and interior reliability within each factor of the PIS-HSP scale. A reliability of 0.7 is a minimally acceptable level of reliability, and 0.8 or greater is preferable [30,31]. From the data collected, the Cronbach’s alpha values for the four factors were 0.96, 0.96, 0.88, and 0.84, and Cronbach’s alpha for the overall scale was 0.95 (see Table 2), indicating that the PIS-HSP scale had fairly satisfactory reliabilities in assessing the participants’ professional identity (see Table 2).

#### 3.2.4. Descriptive Item Statistics and Standard Deviations of the EFA Model for the PIS-HSP Scale

Table 3 shows the average item scores and standard deviations of the EFA model for the PIS-HSP scale.

The participants scored highest on the “professional goals and values” factor (mean = 7.194; 35.94÷5), followed by the “emotional identification and belongingness” (mean = 5) and “professional commitment and devotion” (mean = 5.463) factors. The participants scored lowest on the “self-fulfillment and retention” factor (mean = 4.984).

### 3.3. Confirmatory Factor Analysis (CFA)

#### 3.3.1. Goodness of Fit of the CFA Model for the PIS-HSP Scale

**Figure 2:**
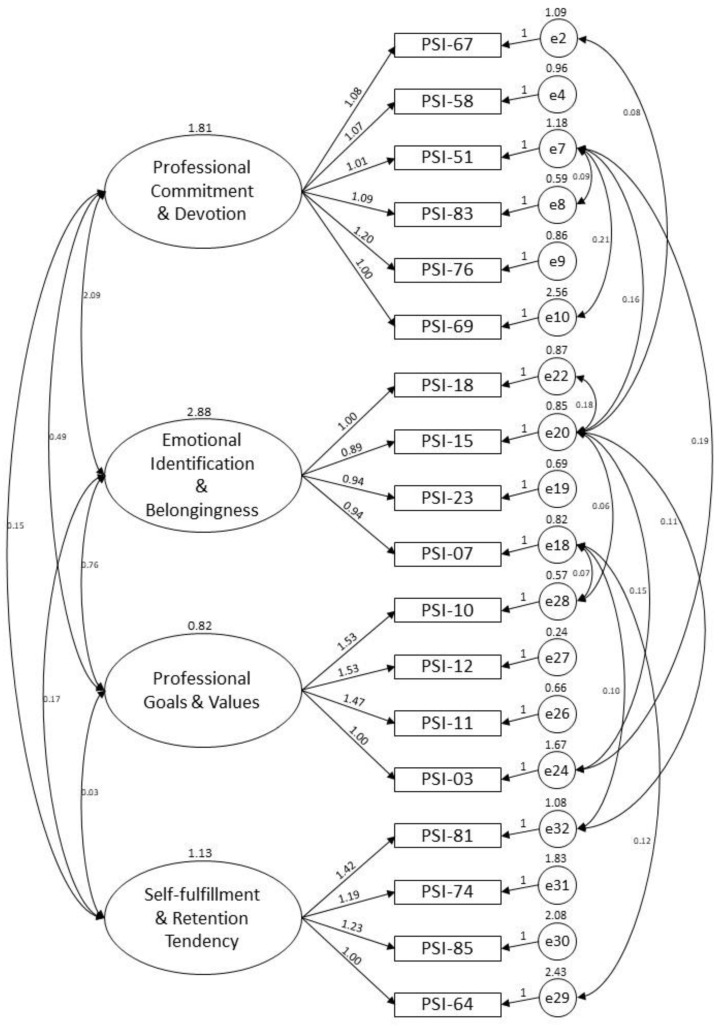
CFA model for the PIS-HSP scale.

In order to evaluate the goodness of fit of the four-factor structure derived from the baseline EFA model, the study further used confirmatory factor analysis (CFA) to examine the underlying latent variable structure of the PIS-HSP scale from the same sample on which the EFA was administered. The CFA model yielded 18 items related to the four factors of “professional commitment & devotion” (6 items; factor loadings: 0.644–0.886), “emotional identification & belongingness” (4 items; factor loadings: 0.854–0.888), “professional goals & values” (4 items; factor loadings: 0.573–0.943), and “self-fulfillment & retention tendency” (4 items; factor loadings: 0.563–0.822). Figure 2 shows the CFA model.

To evaluate the goodness of fit of the CFA model for the PIS-HSP scale, multiple fit indices were used to examine the adequacy of the model’s fit to the data, including the χ^2^/df ratio, Tucker Lewis Index (TLI), comparative fit index (CFI), and root mean square error of approximation (RMSEA). The chi-square divided by the degrees of freedom (χ^2^/df ratio) refers to the amount of difference between observed and expected values, with values less than 3 and *p*-values > 0.05 indicating little difference between the observed and expected values [32] and with χ^2^/df < 2.0 representing an acceptable model fit [33]. The TLI refers to the discrepancy between the chi-squared value of the hypothesized model and the null model. The bigger the TLI value, the better the fit of the model. Though a TLI value larger than 0.90 is considered an acceptable model fit, 0.95 is considered excellent [34]. The CFI refers to the discrepancy function adjusted for sample size, with a CFI value of 0.90 or greater being an acceptable model fit and 0.95 being excellent [35]. The RMSEA index refers to the standard deviation of the residual (prediction error) in the model, with an RMSEA index smaller than 0.08 being an acceptable model fit and 0.05 being an excellent model fit [35,36]. Table 4 shows the results of the goodness-of-fit indices of the CFA model and the baseline EFA model for the PIS-HSP scale—baseline EFA model: χ^2^/df ratio = 3.374 (*p* = 0.000), TLI = 0.912, CFI = 0.918, RMSEA = 0.068; CFA model: χ^2^/df ratio = 1.138 (*p* = 0.146); TLI = 0.997; CFI = 0.997; RMSEA = 0.016.

#### 3.3.2. Reliability

For the CFA model, the study used Cronbach’s alphas and composite reliabilities (CR) to test the scale’s stability and internal consistency, with values higher than 0.70 considered good [37,38]. The Cronbach’s alphas for the four factors and for the overall scale of the baseline EFA model (33 items) were 0.96, 0.96, 0.88, 0.84, and 0.95, respectively. The Cronbach’s alphas for the four factors and for the overall scale of the CFA model (18 items) were 0.91, 0.93, 0.88, 0.78, and 0.89 respectively; the composite reliability coefficients were 0.92, 0.93, 0.89, and 0.78, and 0.97 respectively. With the values all higher than the minimally acceptable value of 0.70, it can be assumed that the baseline EFA model and CFA model for the PIS-HSP scale have fairly satisfactory reliabilities in assessing participants’ professional identity (see Table 5).

#### 3.3.3. Convergent Validity

To further validate the consistency of the derived model, the study also used average variance extracted (AVE) values to test the convergent validity of the PIS-HSP, with AVE values being equal to or greater than 0.50 and lower than composite reliabilities [37,38]. As shown in Table 6, among the AVE values of the four factors, the AVE value (0.48) of the “self-fulfillment and retention tendency” factor is below, though quite close to, the recommended cutoff point of 0.50 [38]. The “professional commitment and devotion,” “emotional identification and belongingness,” and “professional goals and values” factors show acceptable convergent validities (0.651, 0.760, and 0.679, respectively). All of the AVE values are lower than the composite reliabilities (0.917, 0.927, 0.891, and 0.782; see Table 5 and Table 6).

#### 3.3.4. Discriminant Validity

Discriminant validity was evaluated by comparing the square root values of the average variance extracted (√AVE) with the values of the correlation coefficients (*r*) between factors. Discriminant validity is proven if the √AVE is higher than the *r* between factors [38]. Table 6 presents the results of the average variance extracted (AVE), square root of AVE (√AVE), and matrix of correlations between factors. Table 6 also shows that the criterion is met with respect to the discrimination between “professional commitment and devotion” and “professional goals and values” (√AVE = 0.806 and 0.824, respectively; *r* = 0.398), between “professional commitment and devotion” and “self-fulfillment and retention tendency” (√AVE = 0.806 and 0.690, respectively; *r* = −0.089), between “emotional identification and belongingness” and “professional goals and values” (√AVE = 0.871 and 0.824, respectively; *r* = −0.477), between “emotional identification and belongingness” and “self-fulfillment and retention tendency” (√AVE = 0.871 and 0.690, respectively; *r* = −0.092), and between “professional goals and values” and “self-fulfillment and retention tendency” (√AVE = 0.824 and 0.690, respectively; *r* = 0.019). There is a marginal discriminant validity between “professional commitment and devotion” and “emotional identification and belongingness” (√AVE = 0.806 and 0.871, respectively; *r* = 0.832).

The examination of validities and reliabilities shows that the developed CFA model for the PIS-HSP scale and the baseline EFA model can be used as formal scales to measure the subjective sense of professional identity for healthcare students and professionals. The factors and items of the CFA model for the PIS-HSP scale are shown in Appendix A.

## 4. Discussion

This study was intended to develop a scale to measure the professional identity of healthcare students and professionals and to help them acclimate to the interprofessional healthcare system, hence, interpreting or reinterpreting their existence and meaning in their interprofessional identification process. The study first used the EFA to get a longer and preliminary version of the PIS-HSP scale, which yielded 33 items and four factors, accounting for 68.59% of the variances. In the study, the researchers used the varimax rotation to obtain the greatest possible variance, which generally lies in the first factor [29]. The four factors were “professional commitment and devotion” (16 items), “emotional identification and belongingness” (7 items), “professional goals and values” (5 items), and “self-fulfillment and retention tendency” (5 items). The average item scores on the four factors reveal that the participants agree with the values of healthcare and believe that their profession contributes to society. However, they sometimes feel exhausted and have a negative perception of self-worth in response to the current healthcare situation. The factor loadings on each single factor of the PIS-HSP were in the range between 0.61 and 0.89. According to Hair et al. [37], factor loading values above 0.50 are considered adequate, and those above 0.70 are considered good. Hence, it can be suggested that each item is adequate or good for measuring the factor. The Cronbach’s alphas for the four subscales and the overall scale were in the range between 0.84 and 0.96.

Further, the study used the CFA to examine the fitness of the model extracted by the EFA and the underlying latent variable structure of the PIS-HSP scale; the study reduced the preliminary 33 items to 18 items, removing items 66, 84, 61, 63, 43, 48, 79, 37, 50, and 33 in factor 1; items 16, 14, and 21 in factor 2; item 19 in factor 3; and item 73 in factor 4. As a result, a shorter version of the PIS-HSP scale was developed, with only 18 items: professional commitment and devotion (6 items), emotional identification and belongingness (4 items), professional goals and values (4 items), and self-fulfillment and retention tendency (4 items).

The factor loadings for each item ranged between 0.563 and 0.943, suggesting acceptable factor loading values [37]. Hence, it can be demonstrated that each item in the CFA model is an adequate to good indicator of the factor. As for the evaluation of the goodness of fit, while using the multiple fit indices to examine how well the model fit the data, the indices of the baseline EFA model show acceptable model fits in TFI (0.912), CFI (0.918), and RMSEA (0.068), but not in the χ^2^/df ratio (3.374; *p*-value = 0.000). In the CFA model, the model fits in TFI (0.997), CFI (0.997), and RMSEA (0.016) increased from “acceptable” to “excellent.” The χ^2^/df ratio (1.138; *p*-value = 0.146) was acceptable. Hence, it can be said that the PIS-HSP scale has good or even excellent model fit and, hence, has an appropriate and reliable factor structure [36,37,38,39].

In terms of convergent validity, among the four factors, the AVE values of the “professional commitment and devotion,” “emotional identification and belongingness,” and “professional goals and values” factors were above the cutoff value of 0.5 and below the composite reliabilities. However, the AVE value of the “self-fulfillment and retention tendency” factor was 0.477, with a composite reliability value of 0.782. Though the AVE value 0.477 is not above the cutoff of 0.5, with the composite value above 0.6 the convergent validity is acceptable. According to Fornell and Larcker [39], if the AVE value is lower than 0.5, but the composite reliability value is above 0.6, the convergent validity is confirmed.

Turning to discriminant validity, based on the Fornell-Larcker criterion [39], the square root values of the AVE for each factor should be higher than the correlation between the factors. Based on the criterion, the results proved the discriminant validity between the “professional commitment and devotion” and “professional goals and values” factors, between the “professional commitment and devotion” and “self-fulfillment and retention tendency” factors, between the “emotional identification and belongingness” and “professional goals and values” factors, between the “emotional identification and belongingness” and “self-fulfillment and retention tendency” factors, and between the “professional goals and values” and “self-fulfillment and retention tendency” factors. There is a marginal discriminant validity between the “professional commitment and devotion” and “emotional identification and belongingness” factors. One explanation for the derived marginal discriminant validity could be that although the sample size (*N* = 509) was acceptable to ensure a meaningful and sound evaluation, the size might not be big enough to eliminate bias [40]. According to Hair et al. [37], the sample size should be at least five times the number of items used in the scale (a 5:1 ratio). A more acceptable sample size should be ten times the number of scale items (a 10:1 ratio). In the study, the sample size (*N* = 509) met the minimum criterion of sample size 435 (5 times 87 items), though not reaching the more acceptable size (10 times 87 items).

The reliability of the CFA model for the PIS-HSP scale was established by calculating Cronbach’s alpha and composite reliability statistics. According to Cunha et al. [41] and Fornell and Larcker [39], the value between 0.8 and up to 1.0 is considered very high. The minimally acceptable level of reliability is 0.70 [30,31], because all the Cronbach’s alphas values were higher than 0.70, some even higher than 0.90. Thus, the internal reliabilities of the subscales and the overall scale are considered good [37,38,39,41]. Based on the above validity and reliability examination, the research results support the four-factor structure, consistent with the baseline EFA model for the PIS-HSP scale, as well as good internal consistency.

In light of these findings, the developed CFA model and the baseline EFA model for the PIS-HSP scale can be used as formal scales to measure professional identity for healthcare students and professionals, with the baseline EFA model being the long form and the CFA model being the short form.

## 5. Conclusions

This study was intended to develop a comprehensive and psychometrically adequate professional identity scale for healthcare students and professionals based on Taiwanese medical and healthcare contexts. This study provided evidence for the reliability, construct validity, convergent validity, and discriminant validity of the four factors of the PIS-HSP scale. Therefore, the scale is suitable for assessing healthcare students’ and professionals’ professional identity. However, despite the satisfactory results, the present study was based on participants’ self-reports and, hence, might have inherent limitations if the respondents did not provide honest answers. The sample size may also not be big enough to generalize the results, with one marginal discriminant validity between two factors. In addition, because the development of the PIS-HSP was based on Taiwanese cultural contexts and was intended for healthcare students and professionals, those who would like to use the scale for professional identity assessment should consider differences in cultural and professional backgrounds. Future studies may consider using a larger sample across the country to further verify the feasibility of the developed instruments, both the baseline EFA model and the CFA model.

## Figures and Tables

**Table 1:** Participants’ demographic characteristics.

Characteristics	Categories		Number	Percentage
Gender	Male		170	33.40%
	Female		339	66.60%
Source	Healthcare professionals		133	26.13%
	Students	College of Medicine	129	25.34%
		College of Medical Science and Technology	182	35.76%
		College of Health Care and Management	65	12.77%
Age	19 + to 22 years old		320	62.87%
	22 + to 30 years old		111	21.81%
	30 + years old		78	15.32%
Educational background	High school		34	6.68%
	University		367	72.10%
	Graduated school		108	21.22%
Ever married	No		389	76.42%
	Yes		120	23.58%

**Table 2:** Rotated factor loading of the EFA model for the PIS-HSP.

Item	Factor 1: Professional Commitment & Devotion	Factor 2: Emotional Identification & Belongingness	Factor 3: Professional Goals & Values	Factor 4: Self-Fulfillment & Retention Tendency
Factor 1: *α* = 0.96				
66	0.82			
67	0.76			
84	0.75			
58	0.73			
61	0.72			
63	0.71			
51	0.70			
83	0.70			
76	0.68			
69	0.68			
43	0.67			
48	0.66			
79	0.65			
37	0.64			
50	0.61			
33	0.61			
Factor 2: *α* = 0.96				
16		0.79		
18		0.75		
14		0.74		
15		0.73		
23		0.67		
7.		0.64		
21		0.64		
Factor 3: *α* = 0.88				
10			0.89	
12			0.89	
11			0.84	
19			0.68	
3			0.65	
Factor 4: *α* = 0.84				
73				0.88
81				0.83
74				0.78
85				0.78
64				0.64
Eigen value	15.62	3.21	2.70	1.12
% of variance	47.33	9.72	8.17	3.38

Overall *α* = 0.95; total variance explained is 68.60%.

**Table 3:** Average item scores and standard deviations of the exploratory factor analysis (EFA) model for Professional Identity Scale for Healthcare Students and Professionals (PIS-HSP).

Item	Mean	S.D.
Factor 1: Professional Commitment & Devotion	**87.40**	**23.11**
66. Even if the salary is not satisfactory, I (will) remain in healthcare work.	4.78	1.79
67. Even if I am married, I (will) remain in healthcare work.	5.46	1.80
84. I like healthcare work. Even if there are other job opportunities with higher pay, I will not consider them.	5.00	1.97
58. I would regret it if I have to leave healthcare work.	5.41	1.75
61. If I could choose my career again, I would still choose to work in healthcare.	5.49	1.87
63. I (will) devote all my time to healthcare work, even if I have to sacrifice time with my family.	4.26	2.09
51. No matter how many setbacks I may receive in healthcare work, I will never give it up.	5.53	1.75
83. I like my healthcare specialties and am actively preparing myself for the profession.	5.69	1.66
76. My personal goals are highly relevant to my healthcare work.	5.62	1.86
69. I often read journals and books related to healthcare work to enrich my professional knowledge.	5.65	2.09
43. I often think of my healthcare work that I am responsible for right now, as well as the work that I have not yet finished and completed.	5.46	1.83
48. My personality and personal beliefs are consistent with the characteristics and values of healthcare work.	5.65	1.77
79. I think healthcare work itself is challenging and stimulating.	6.10	1.72
37. I am always thinking about how to do better in healthcare.	5.68	1.70
50. I believe I can succeed in a healthcare career.	5.87	1.78
33. Engaging in healthcare work gives me psychological satisfaction.	5.74	1.85
Factor 2: Emotional Identification & Belongingness	**40.54**	**11.58**
16. For me, healthcare is the best career that I can do.	5.49	1.95
18. I am proud of being a healthcare professional.	5.74	1.94
14. Even if there are job opportunities other than healthcare, I (will) persist in being a healthcare professional.	5.52	1.93
15. It is important for me to devote myself to healthcare work.	5.91	1.79
23. I have a strong interest in healthcare and always enjoy working in this field.	5.73	1.81
7. I am happy with choosing healthcare as my profession.	6.17	1.83
21. I feel that I am a member of the healthcare profession.	5.98	1.79
Factor 3: Professional Goals & Values	**35.97**	**6.53**
10. I think that healthcare is a professional job.	7.43	1.58
12. I agree with the value of healthcare work.	7.39	1.46
11. I think that healthcare is a respected profession.	7.30	1.56
19. I am sure that the healthcare workers are interested in helping people.	6.94	1.72
3. I believe that healthcare is a profession that can bring certain contributions to the country and society.	6.91	1.58
Factor 4: Self-Fulfillment & Retention Tendency	**24.92**	**7.41**
73. I regret choosing healthcare as my career. (-)	5.05	1.93
81. I often want to change my current job. (-)	4.99	1.84
74. If I have the chance to choose, under the same working conditions, I will choose a job that is not related to healthcare. (-)	4.89	1.86
85. I have a negative perception of self-worth in response to the current healthcare situation. (-)	5.08	1.95
64. Healthcare is only one of the many jobs I can do; I cannot put my whole heart into it. (-)	4.91	1.88

The values in bold refer to factors' mean and S.D.

**Table 4:** Goodness-of-fit indices for the baseline EFA model and the confirmatory factor analysis (CFA) model for the PIS-HSP scale.

	$\chi^{2}$	df	χ2df	*p*	TLI	CFI	RMSEA
Baseline EFA Model	1649.885	489	3.374	0.000	0.912	0.918	0.068
CFA Model	133.157	117	1.138	0.146	0.997	0.997	0.016

Note: df = degree of freedom; TLI = Tucker Lewis index; CFI = comparative fit index; RMSEA = root mean.

**Table 5:** Cronbach’s alphas and composite reliability coefficients of the baseline EFA model and the CFA model for the PIS-HSP.

Reliability Factor	Cronbach’s Alpha PIS-HSP: Baseline EFA Model and Long-Version Scale	Cronbach’s Alpha PIS-HSP: CFA Model and Short-Version Scale	Composite Reliability PIS-HSP: CFA Model and Short-Version Scale
Professional commitment & Devotion	0.96	0.91	0.92
Emotional Identification & Belongingness	0.96	0.93	0.93
Professional Goals & Values	0.88	0.88	0.89
Self-fulfillment & Retention Tendency	0.84	0.78	0.78
Overall	0.95	0.89	0.97

**Table 6:** Average variance extracted (AVE), square root of AVE (√AVE), and matrix of correlations between factors.

Factor	AVE	1	2	3	4
1. Professional Commitment & Devotion	0.651	**0.806**			
2. Emotional Identification & Belongingness	0.760	0.832 **	**0.871**		
3. Professional Goals & Values	0.679	0.398 **	0.477 **	**0.824**	
4. Self-Fulfillment & Retention Tendency	0.477	−0.089 *	−0.092 *	0.019	**0.690**

The values shown in bold are the square root of AVE (√AVE) ** *p* < 0.001; * *p* < 0.05.

## Appendix A. The Factors and Items of CFA Model for the PIS-HSP Scale

Factor 1: Professional Commitment & Devotion

67. Even if I am married, I (will) remain in healthcare work.
58. I would regret it if I have to leave healthcare work.
51. No matter how many setbacks I may receive in healthcare work, I will never give it up.
83. I like my healthcare specialties and am actively preparing myself for the profession.
76. My personal goals are highly relevant to my healthcare work.
69. I often read journals and books related to healthcare work to enrich my professional knowledge.

Factor 2: Emotional Identification & Belongingness

18. I am proud of being a healthcare professional.
15. It is important for me to devote myself to healthcare work.
23. I have a strong interest in healthcare and always enjoy working in this field.
7. I am happy with choosing healthcare as my profession.

Factor 3: Professional Goals & Values

10. I think that healthcare is a professional job.
12. I agree with the value of healthcare work.
11. I think that healthcare is a respected profession.
3. I believe that healthcare is a profession that can bring certain contributions to the country and society.

Factor 4: Self-fulfillment & Retention Tendency

81. I often want to change my current job. (-)
74. If I have the chance to choose, under the same working conditions, I will choose a job that is not related to healthcare. (-)
85. I have a negative perception of self-worth in response to the current healthcare situation. (-)
64. Healthcare is only one of the many jobs I can do; I cannot put my whole heart into it. (-)
